# How police officers juggle work, a life partner, and kids

**DOI:** 10.3389/fpsyg.2023.1178314

**Published:** 2023-07-06

**Authors:** Elin Granholm Valmari, Ulla Nygren, Mehdi Ghazinour, Kajsa Gilenstam

**Affiliations:** ^1^Occupational Therapy Unit, Department of Community Medicine and Rehabilitation, Umeå University, Umeå, Sweden; ^2^Police Education, Umeå University, Umeå, Sweden

**Keywords:** daily hassles, equality, gender, life balance, lifestyle, patrol service, role balance, qualitative content analysis

## Abstract

Police officers frequently encounter stressful social situations during their working days. Furthermore, previous research on policing and families show that police officers’ families are impacted in different ways when at least one member of the family has the role of a police officer. Despite work spilling over to family life there is currently little research on police officers’ role-balancing. Thus, the purpose of this study was to explore and describe the challenges that arise at the intersection between police officers’ professional roles and their private life roles as parents and life partners, as well as how police officers balance these roles in between. We used qualitative content analysis after interviewing 13 uniformed police officers. The findings show how the police officers’ professional roles affect their private life roles within three different sub-themes and are summarized under the theme of “Balancing conflicting roles: Coping with professional and private life commitments”. The theme revolves around the various challenges of working as a uniformed police officer, such as hypervigilance and risks, as well as the enrichments and conflicts of working shifts while also juggling private life roles. The results also touch on gender and equality in life-partner relationships. The study raises an important question about how these challenges can be mitigated within Police authorities to enable uniformed police officers to balance their professional and personal lives in a healthy and sustainable manner.

## 1. Introduction

Uniformed police officers—officers interacting with the public daily, responding to emergency calls, or patrolling specific areas on foot or in a vehicle while keeping the public safe and upholding the law (the Riksdag, 1998; PATROL OFFICER, 2022)—frequently engage in challenging contexts and environments while at work. The different features of their social environments at work, such as adequacy of communication or emotional support and collaboration, impact the police officers’ life and health (Granholm Valmari et al., 2022b). Their physical environment at work can also include risky and traumatic work activities throughout the course of their working day (Mona et al., 2019). Both social and physical environments at work may also provide both barriers and resources for the police officers’ balance in life (Granholm Valmari et al., 2022a) and affect their personal lives (Qureshi et al., 2019).

Previous research on policing and family issues has been summarized in a prior review (Violanti et al., 2017), indicating that police officers’ marriages and families are impacted in different ways when at least one member of the family has the role of a police officer (Miller, 2007; Tuttle et al., 2018). Hence, being a police officer may lead to stress spilling over to family life, challenging the social relationships at home (Tuttle et al., 2018). For example, Canadian researchers have found police officers taking work home with them (Duxbury and Halinski, 2018). Furthermore, they struggle to maintain a healthy work-family balance while caring for their elderly and children as well as working full-time (Duxbury et al., 2021). A Swedish research report also shows that police officers worry that their job will affect their families’ health, such as their loved ones being threatened (Sundqvist et al., 2021). Being a police officer may also result in secondary victimization, where spouses can be affected by trauma experienced by their significant other (Friese, 2020). Conflicts between work and family life for police officers have also been linked to stress and mental ill-health in both Norway (Mikkelsen and Burke, 2004) and India (Lambert et al., 2017; Qureshi et al., 2019). Additionally, it has been demonstrated that police officers who experience work-life conflict are more likely not to recover mentally after their shifts (Elgmark Andersson et al., 2013).

## 2. Roles in life, role conflict, and role balance

The interface between work and family, including roles, has been researched in various ways. Role balance (Marks and MacDermid, 1996), work-family role conflict (Greenhaus and Beutell, 1985), or work-family spillover (Bakker and Demerouti, 2013) are some examples. Roles organize how we behave and relate to others, as well as shape what we do. Roles also divide our daily and weekly cycles into time periods when we inhabit certain roles. During our days, and within our social environments, roles overlap, and some roles involve a succession of other roles. A person may also have several roles in the same social environments, which occupy the person’s routines, time, and space (Taylor, 2017; Wook Lee and Kielhofner, 2017). Hence, in the workplace, a person is mainly in the worker role, and at home, for example, has the role of parent or life partner. Having complementing roles gives rhythm and change between different identities and modes of doing things in life (Taylor, 2017).

Roles are also affected by culture and gender (Taylor, 2017), indicating that gender-related patterns affect life partner relationships (Connell, 2021). For instance, for men, male stereotypical behavior, such as being aggressive or managerial at work, may conflict with exhibiting the same behavior at home because family members expect different behaviors from them in their private lives compared to their professional lives, such as being nurturing (Greenhaus and Beutell, 1985). Having a more traditional gender role attitude such as the belief that women should perform household chores and men are the main breadwinners, also result in lower well-being as well as a higher work–family conflict for both men and women (Chen et al., 2022). Additionally, for men but not for women, stress-based work–family conflict may be lowered by life partner support (Adams and Golsch, 2021).

According to Van der Lippe et al. (2004) Sweden has taken the lead in promoting gender equality within the police force, for example implementing government policies such as gender mainstreaming and reaping benefits from publicly funded childcare. Furthermore, work-family arrangements, such as working part-time or offering flexible working hours and replacement while on leave, has been important for gender equality within the Swedish police force (Van der Lippe et al., 2004). Despite of this, a Swedish research project has shown gender inequalities regarding health between men and women. Male patrolling police officers had a 1.2 likelihood of better mental recovery after work than female police officers (Elgmark Andersson et al., 2013). One reason for the complexities of role conflict for both male and female uniformed police officers may be that they work in a male-dominated organization (Duxbury et al., 2021). Furthermore, Haas and Hwang (2019) concluded that flexible work practices in male-dominated organizations are not possible due to workplace culture and work structure despite Sweden’s efforts to promote gender equality. Thus, there is an expectation of fathers working and not taking parental leave, regardless of Swedish policy requiring employers to allow fathers to take parental leave (Haas and Hwang, 2019).

Having different roles in life also necessitates balancing them, which could entail investing more time or attention to certain roles. Another example of balancing roles is to even-handedly allocate personal resources among the different roles to balance one’s life (Marks and MacDermid, 1996). Hence, in the same way that the professional role may collide with private life roles, they can also enrich one another by transferring resources or emotions from one role to the next (Greenhaus and Powell, 2006). Role balance is the result of ongoing adjustments to priorities and activities within various roles, including both enrichment and conflict between roles, and is, therefore, both dynamic and complex (Evans et al., 2014). According to Greenhaus and Beutell (1985) there are three sources of stress in terms of role conflict: time-based conflict, strain-based conflict, and behavior-based conflict. According to Marks and MacDermid (1996), people with more balanced role systems will also report less role strain, greater role comfort, and better well-being than those with a less balanced role system. Role balance has also been found to be related to self-esteem (Marks and MacDermid, 1996), as well as to be important for health and well-being (Moll et al., 2015). This is also why for example the European Foundation for the improvement of Living and Working Conditions (Eurofound), are concerned with, and strategically work towards work-life balance as a way to raise the bar on job quality (Eurofound, 2019, 2023). However, when a person cannot meet the obligations or aspirations represented in several roles within their lifestyle, role strain occurs (Kielhofner, 2008). Role imbalance is therefore opposite to role balance (Evans et al., 2014).

According to Perreault and Power (2023) work and life have been treated as two separate domains within organizational psychology. Work has been regarded to be on one side and equal to everything else in life, indicating work to be of primary importance. Balance is regarded to occur in-between these two domains. However, role balance has been suggested to better explain between-role conflicts when utilizing an individual perspective instead of an organizational perspective (Perreault and Power, 2023). According to Marks (2014) role balance is also a gendered issue. For instance, men feel less balanced the more they work, whereas women feel less balanced the more time they spend with their children (Marks, 2014). A longitudinal study in Sweden also showed that work–family conflict was related to an increased risk of poor health for women, while for men there was an increased risk for problem drinking. Thus, men and women’s health is affected in different ways by their professional role affecting private life roles (Leineweber et al., 2013). Moreover, a Swedish research report discovered police officers working in vulnerable areas to be dissatisfied with their life partner if they also experienced high levels of operational stress at work (Sundqvist et al., 2021). Hence, despite existing research on police officers’ contexts and environments (Mona et al., 2019; Granholm Valmari et al., 2022b), work-life conflicts (Duxbury and Higgins, 2012), and role conflicts (Wu, 2009), we are not aware of any earlier research on police officers’ role balance from an individualistic perspective, where professional and private life roles are included on similar terms. Furthermore, since role balance also affects health (Marks, 2014), and the police profession is a male-coded organization (Duxbury et al., 2021), role balance of police officers warrants further investigation. Moreover, there is relatively little research on the challenges police officers face in both their professional and private life roles and how they balance their roles regarding these challenges. Consequently, we sought to gain a better understanding of how uniformed police officers balance their roles between work and private life. Accordingly, this study aims to explore and describe the challenges that arise in the intersection between the police officers’ professional roles and private life roles of being a parent and partner, and how the police officers balance these roles.

## 3. Method

The approach of conducting semi-structured interviews was selected for its capacity to increase understanding of the experiences of uniformed officers’ role balance. Graneheim and Lundman’s (2004) qualitative content analysis methodology includes a variety of ways of analyzing the data, from describing similarities and differences to describing a red thread within the data and illuminating themes of meaning (Lindgren et al., 2020). Therefore, their method was chosen to answer the study’s aim of exploring these police officers’ balance between roles.

To ensure transferability, the methods, and results (including quotations) were described in detail according to the Qualitative Design Reporting Standards (JARS-Qual) (Levitt et al., 2018). JARS-Qual has also been for performing the study and writing the paper. Ethical approval for this study, as part of a larger research project, was obtained from the Swedish Ethical Review Authority 2020–07170.

### 3.1. Participants and procedure

The dataset includes 13 uniformed police officers from Sweden working within different patrol services, for example, focusing on emergency response, road, or community policing duties. These participants are not only police officers but also parents and current or ex-life partners. The dataset is part of a larger dataset aiming to understand police officers’ lifestyles and health. After analyzing the data from 13 participants, the results reached the point of information power (Braun and Clarke, 2022). Consequently, the author group decided no further data needed to be collected.

**Figure 1:**
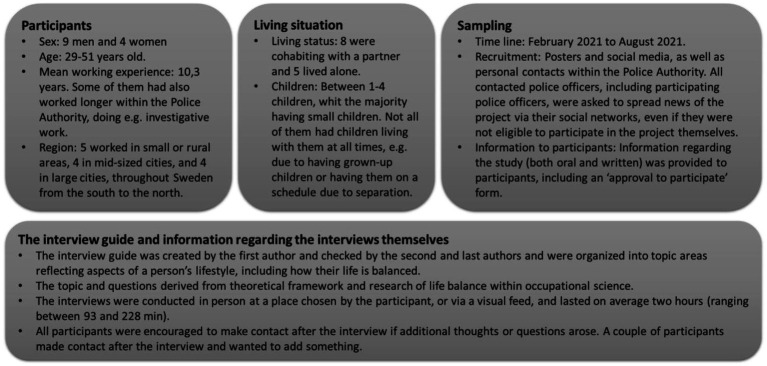
Information regarding participants and sampling procedure.

To ensure the adequacy of data, both purposive as well as snowball sampling were used to gain a rich dataset of police officers’ experiences regarding balancing their roles (gender, family situation, living and working in an urban or rural area, as well as locations in Sweden); see Figure 1 for the sampling procedure as well as more participants’ characteristics.

The first author conducted and audio-recorded all interviews. An interview guide, see Figure 1, was used in the first few interviews, but after that, the participants’ narrative steered the conversation, as well as the order of topics and questions. Questions from the interview guide were added if the participants did not address the various topics themselves. The interviewer strived to encourage a relaxed atmosphere to make the participants feel comfortable and receive their undivided attention. More information regarding the interview and interview guide is found in Figure 1.

Due to the sensitive nature of the content, the interviews were transcribed verbatim by professional transcribers, the majority of them by a medical secretary. The first author read the transcripts and checked for any deviation between the recordings and the transcripts. For reasons of confidentiality, identifying details were omitted or altered, and fictitious names will be used for presenting the findings.

### 3.2. Data analysis

Qualitative content analysis offers a systematic process for interpreting qualitative data, by focusing on expressions of experiences while identifying similarities and differences in manifest and latent data by going back and forth in the data analysis (Graneheim and Lundman, 2004; Graneheim et al., 2017; Lindgren et al., 2020). The analysis was performed mainly by the first author. The other authors also took part in the analysis, for example by participating in triangulating the analysis, reading manuscripts, locating potential analytic interests, comparing transcripts with the initial analysis, etc. Thus, following the aim of this study and using qualitative content analysis, the data were first decontextualized and then recontextualized according to Graneheim et al. (2017):

- The first author located the already de-contextualized data consisting of condensed meaning units that had been abstracted in a previous study. Then, both the manifest and latent meaning in the data was labelled with a code, using MAXQDA (VERBI Software, 2019). Most codes were, however, at a high level of abstraction due to the complex phenomenon of balancing roles. Furthermore, all transcripts were read again with the study’s aim in mind to ensure nothing was missed.
- Re-contextualization was done by comparing codes according to “police role and parent” and “police role and life partner” and categorizing them, based on differences and similarities. Then, to make sense of the data despite its richness and high abstraction level, the codes were unified in underlying meanings. Furthermore, interpreting both the manifest and latent content into themes and sub-themes.

## 4. Results

The analysis resulted in one theme, “Balancing conflicting roles: Coping with professional and private life commitments”, and three sub-themes, see Table 1.

**Table 1:** Short description of theme and sub-themes.

**Balancing conflicting roles: coping with professional and private life commitments**		
The theme revolves around the challenges in balancing professional responsibilities with private life roles. The challenges include being torn between professional and private life roles, feelings overshadowed by their demanding profession, being hypervigilant and experiencing guilt. The police officer role can also enrich their private life roles in various ways. The study highlights the importance of building strong life partner relationships, where emotional and social support is crucial, given the demands of their professional role. Overall, the theme sheds light on the unique difficulties faced by uniformed police officers in managing their time, emotions, and life priorities, while fulfilling their passion for police work and meeting their family responsibilities.		
**Finding harmony between professional and private life**	**Navigating life’s priorities while working shifts**	**Building strong relationships through emotional and social support**
This sub-theme centers around how their professional role both enriches and causes conflicts between their professional and parental roles. The professional role often takes priority over the other roles. Examples are hypervigilance in private life due to professional risks, private life roles experienced as less exciting, or stress spilling over to private life.	This sub-theme highlights advantages of working shifts, such as more time with family. It also includes challenges in terms on time management, and how to organize family life due to practical complications of their professional role.	This sub-theme is defined by how the police officer role integrates with private life roles and how they manage their life partner relationships. For instance, building a strong relationship through communication, being sensitive towards their life partner, as well as supporting each other. The sub-theme also includes gender issues and how to achieve equality in a relationship.

The results highlight the significant challenges faced by uniformed police officers in balancing their professional responsibilities with their private life roles. The findings shed light on the unique difficulties experienced in managing their time, emotions, and life priorities. The impact their professional role has on their private life roles includes being torn between work and family commitments, causing conflicts between roles. Hence, the police officers express concerns about their private life feeling overshadowed by their demanding profession, leading to feelings of guilt. Furthermore, the findings highlight how the uniformed police officers adopt behaviors to mitigate risks in their private lives. For example, to avoid running into people and having to intervene as police officers while out with their families, they might, for example, exclude certain leisure activities from their private life roles. Nonetheless, the police officer role also enriches their private life roles in different ways, such as knowing what an actual risk is. For example, since it is their job to assess risks, they also know what an actual risk is instead of worrying about everything. Another example is shiftwork, which provides opportunities during the daytime to spend more time with their families. Additionally, the study presents findings related to navigating priorities in life as they fulfill their passion for police work while meeting their family responsibilities. Adjacent to this is the building of strong life partner relationships, where emotional and social support is important, particularly in the context of the police officers’ demanding professional role.

### 4.1. Finding harmony between professional and private life roles

This sub-theme revolves around balancing the different roles in life, where they sometimes enrich each other, and sometimes are conflicting. The professional role is considered by the police officers to augment their family life and private life roles in different ways. One example was the advantage of understanding the difference between perceived and actual risks concerning danger when, for instance, raising teenagers. Since Lewi knows the city he works in, he also knows where there are more and less risks for teenagers to get into trouble. Thus, he explains that his wife used to worry more than him when their teenagers were out and about in the evenings. Additionally, they worried about entirely different things due to him having more knowledge of risk assessment than her. His professional role also enriched his parental role in another way: *‟I’ve had a, shall we say, well-functioning intelligence service regarding the whereabouts of my kids, where they have been and in what contexts and things like that. So, both I and my wife have received feedback directly from my colleagues there ….”* The parental role also enriches the strains caused by their professional role. Lucas elaborates on how children can ease bad feelings when returning home from a long day at work with unpleasant work tasks: *“…children are wonderful…when you have had a…bad, tough day…you come home, and you are greeted by happy children.”* Rebecca, who has small children confirms this, but also adds how the parental role can be tiring, even more so than the professional role: *‟… when I’m at work … well I feel like I’m really recovering from home. What stresses me out the most in my life and when I’m at home I recover from work … it’s two different worlds so I feel like I’m taking a break. Being at home for a very long time makes me really tired. Like now for the whole summer, I was more tired than I am now when I’m working.”* Enrichment between roles is one aspect of how roles can be balanced in between. Conflicting roles is another aspect of role balance, where stress from one role spill over to another, such as the police officer role spilling over to the role of parent or life partner. This can manifest in a variety of ways, such as for Samuel who describes how he feels when he is unable to hold it together at home: *“When I was young, I punched many holes in the wall as I often got angry. Not anymore. Now I get mostly sad when I’ve had a bad day. Many get angry, resulting in family problems, but I usually get sad.”*

Another aspect of parenting is finding oneself in a different phase of life, which means that suddenly one’s own needs must be set aside because of children and family. This is not always easy to combine with the demands of their police officer role, such as always being fit. This specific demand is usually put upon themselves to function physically at work. However, the activity must be performed within their private life roles due to the limited time to work out during work hours. Hence, when other things in life take over, such as taking care of children and household chores, the demands of their professional roles must take the back seat, although staying fit has not become less valuable. Consequently, the time within private life roles is prioritized. For some of these police officers, this means that the number of roles they have time and energy for is limited to being a police officer, a parent, a life partner, and maybe a friend to somebody. As a result, previous roles, such as youth sports trainer, are now on hold. Another way for these police officers to juggle their priorities in their private life roles is to multitask. They take kids with them while exercising or combine exercise time with being on the way to work. To be able to fit everything into their private life roles they constantly evaluate and prioritize activities to balance the demands of both their professional role as well as their private life roles. Hence, the parental role was found both conflicting and enriching their professional role.

Another aspect of role balance is if one role takes priority over other roles. For the uniformed police officers in this study, this happens in relation to their hypervigilance. Hence, they avoid and limit their activities in private life roles due to the risk of what might happen. The hypervigilance which comes with their professional role and spills over to their private life roles is explained by Samuel this way: *“I do not like going out to eat in town with my wife, for example … I always walk a few meters ahead of my wife, for example, so that we do not walk together, and I’m on my guard. So, I avoid things like that ….”* Police officers also generally choose where to live and work more cautiously, especially after having children. For example, they may choose not to work in the same area as where they live or specifically choose living areas they know are more crime-free than other areas. As Rebecca puts it: *“And cops usually live in areas where you do not make arrests on your neighbors.”* Thus, another pertinent concern is that the police officers do not always feel safe in their private life roles, because of their professional role. They worry that their life partners or children will be innocently affected. Other conflicting challenges were also raised, such as the feeling of not being compensated adequately for what they do and the danger they face, or the time they spend away from their parental or life partner roles.

The professional roles also take priority in other ways, such as the feeling that everyday life pales in comparison to the professional role. For example, Samuel feels he is doing a good job in his police officer role, where he also receives praise. Praise at work is also more rewarding to him than praise from his children, even though saying this makes him feel ashamed. He explains that being a police officer is a drug that he cannot get off. Another example is from Daniel, who explains his feelings at home when he is in his parental role: *“… it is a bit slow … these everyday things like hanging around the house, reading the same books, taking a stroll in the garden and things like that. But of course, I’m glad that they are feeling well, when you notice that they are happy.”* To be able to understand this contrast in how everyday private life roles can feel so boring, Levi offers an explanation comparing the police profession to other professions: *“… the adrenaline rushes are actually what make this job so special … Here we are still talking about this old classic ‘life and death’ … it becomes so tangible, and that’s probably what I still find fun about this job, that it is real.”* He also elaborates on why he cannot switch to working dayshifts only, even though his wife wants him to: *“… It’s a job that offers freedom, and just the uncertainty. When I go to work, I do not know where I will end up, I do not know what will happen, and to some extent, I do not know when I will get home. So that’s probably the attraction anyway.”*

### 4.2. Navigating life’s priorities while working shifts

This sub-theme is defined by how the uniformed police officers describe how working shifts as a police officer has its pros and cons. Many of the police officers believe that working shifts gives them an advantage when they have small children. They can take their children swimming or to indoor playgrounds while most people are at work and there are fewer people where they want to go. If the other parent is on parental leave, they can also have more family time during the week. Financial difficulties and the feeling of not earning enough were also addressed as a male concern in the study. Consequently, working shifts and deciding to take on extra shifts, was an enrichment to the male police officers regarding private life in terms of providing for their family.

A conflict emanating from shift work impacting their private life roles was regarding time management. The police officers frequently had to solve problems and adapt to collisions in life due to shift work and overtime. For example, Wyatt feels stressed over doing unimportant work assignments as a part of his job, while working involuntary overtime, which collides with his practical responsibilities of being a father and life partner: *“… you have a case that takes up very, very much time, it might not really be a difficult matter, but there is simply too much to write and you just want to go home. Then I can feel that it gets very difficult because I feel that the wife is tired, my boy misses me, I want to go home, I do not feel like sitting here writing this crap right now … shit, I know I have to pick up from kindergarten, I cannot get hold of my wife. Then I get stressed, and it’s really quite taxing.”* Thus, not knowing exactly when a shift will be over creates stress for some of the police officers. Especially if they must leave their children somewhere and do not have any relatives who live nearby. If their life partner works during the day and is responsible for picking children up from for example kindergarten, this is made easier. As well as having relatives close by who can help with childcare when needed. Then it is less stressful to find someone to cover for them when their shifts conflict with their parental role. This uncertainty also conflicts with their life partner role. Due to their professional role, it is not always clear that they can get off work when they are supposed to. Rebecca’s life partner is also a police officer, and even though she can relate to her husband not getting off shift on time, she still explains it like this: *“So you can never rely on … we have a party that starts at five and you finish at four, but at a quarter to four, someone is about to kill themselves. Then you cannot say, hey you, my shift is over now, bye. But then you stand there until eight o’clock until the person feels better. And then you will miss everything. And things like that, I can imagine that people who are not police officers would probably have been quite disappointed...you feel a little abandoned.”*

Another practical complication is when the family is home during the daytime and the police officer needs to sleep. Then resting and sleeping becomes a challenge also to their life partner who must *“carry out a sleight of hand”* to juggle kids as well as a sleeping husband, as Levi puts it. Another issue is that not all municipalities have daycare where children can be left overnight. The police officers feel that the Police authority should aid in the transition after having children, to maximize the possibility of continuing to work as a uniformed police officer. Not only could the Police authority assist, but municipalities also need to provide childcare during the night.

An additional challenge is working shifts and being in a life partner relationship where both partners work shifts. Sophie gives a personal example of how her ex-life partner used to work shifts, even though he was not a police officer himself: *“… when we looked at our schedules … which were a two-shift and my three-shift schedule, it was like someone had put them in overlap. So that if I worked Monday, Tuesday, and the weekend then he worked like Wednesday, Thursday, Friday, or Tuesday, Wednesday, Thursday. Er, so that, and then we got help from his mother then … to pick up at kindergarten and sleep over ….”* For Sophie, this resulted in one reason for why she and her life partner separated. Ava who has also separated from her husband elaborates on why she believes overcoming these obstacles and balancing professional and personal life roles is difficult as a police officer: *“… partly that the working hours can consume much more than expected. And then family life comes along and sort of changes the situation and that is why there are many who, after a few years, move on to slightly, what can I say, more comfortable tasks … more comfortable working hours and a slightly more comfortable work environment.”*

Managing conflicts between the professional role and private life roles was not easy and required strategies such as compromising with time or finding smart solutions to juggle the different roles. At some workplaces, the organization also provided some support. Such as flexibility regarding scheduling, or the possibility to agree upon a joint schedule among co-workers who had children. Many of the police officers with a life partner also tried to properly plan what happens during the week with their respective others so that things would run smoothly, for example when picking up kids during the week. For single parents, it was found to be the most challenging, and they had to find alternative solutions to continue working as a uniformed police officer. For Liam, after divorce, he tried to optimize his time with his daughter to suit both his professional role and parental role. He continued working shifts as before and had his daughter every other week. Additionally, he worked weekends every other week. Hence, he was a single parent working one weekend, so he had no time to rest. The following week, when he had his daughter, he had the weekend off, but he was still exhausted because he felt he had not had time to unwind*; “…working the hours that three shifts entail, means they are fixed. They do not correspond so well with dropping off and picking up at preschool and other stuff. So, I tried for a couple of years, but… I went into a stress-related illness after that which … is a consequence of that.”* Jack on the other hand has found another way to continue working as a uniformed police officer after divorce. He has his children only every other weekend. As he puts it: *“I could for example keep them every two weeks, but then I would not be able to have the job I have.”* Hence, he has chosen his professional role over his parental role. Also, if single parents have their children every other week and are always working every other weekend, they must schedule and plan their work around being a single parent. This usually means putting in long hours the week the children are away and taking more time off the following week when the children are back home. Nevertheless, this only works if it is organizationally feasible, and not all of them have scheduling they can influence. As a result, balancing shiftwork and private life roles is especially difficult for those who are separated and have children.

### 4.3. Building strong relationships through emotional and social support

This sub-theme is defined by how the uniformed police officers try to keep life partner relationships sustainable and alive, despite having a demanding profession. Gender inequalities and dividing the workload at home are also addressed in the sub-theme.

Some of the police officers, particularly men, emphasized the importance of having a functioning sexual life, which did not always imply sexual intercourse, but intimacy and communication. Additionally, taking time for mutual activities, or relaxation, together as a family, or together as a couple, was important. It was also crucial to share more things than just their children. Moreover, also the opportunity to spend some time alone. For example, one police officer who shares both her profession and workplace with her longtime partner describes the importance of having something that they do not share. So, for her, not seeing her husband that often due to shiftwork was a way of getting time alone. The police officers also raised aspects such as overcoming both individual differences and being understanding towards each other, as well as showing respect, as important issues to succeed in a relationship. Samuel elaborates on the topic: *“…the key is that you have an understanding and respect for each other’s thoughts and feelings … it probably works differently for different people, but … you still have to find a way to give both space … if I were to compare it to when my wife was pregnant or when we just had a baby, all the focus is on mom, but it will never last in the long run unless dad also gets attention.”*

Striving for equality in the relationship was also touched upon by the police officers. It was found to be a way to build a strong relationship and overcome inequality and gender differences. For example, Mia is in a heterosexual relationship with another uniformed police officer, and both work shifts. She explains: *“… family-wise, I feel that it is pretty much me who sacrifices to make it work.”* Thus, there is a distinction between male and female roles. Female police officers believe they must shoulder more responsibility for the family and that their life partners’ professional roles usually take precedence. This kind of inequality led Ava to divorce her husband. She felt that things were better after the divorce because they shared the responsibilities better when the children lived with them for a week in turn. She explains the reason for her separation like this: *“That he had his own business and worked a lot and I took a lot of responsibility at home … which eventually led to a separation.”* Thus, some of the police officers had changed life partners during their years working as police officers or were on the verge of separating. Several reasons for why were specified, but they centered around a lack of communication and gender inequalities in the relationship. Most men, also those that were still in a life partner relationship, admitted to their life partner taking a greater responsibility regarding family. Female police officers also agreed that they often did more when it came to family and household. Additionally, the male police officers explained how they tried to do their part to unload family work from their life partner. However, it was more in terms of “helping out” than sharing the load. As Ethan describes it: *“… my partner, she is on parental leave and at home, and … I kind of feel that now I have to go home because I need to sort of relieve her….”*

This inequality makes the male police officers feel as though they have a debt of gratitude that must be paid when they get home from work. It also inflicts a bad conscience on the men to perform better at home and feel inadequate in their private life roles. As Lucas puts it when talking about the difficulty of being enough as the *‛great challenge’*: *“that I can feel that I’m not quite enough, that I cannot make it work. It is important for me to get this exercise that I talked about [as a police officer], it is important that you perform at work, at home you must perform. It can make me stressed … that I do not really feel like I can make it work.”* For the male police officers, it was also more about the sense of validation, than their life partner carrying the heavier burdens of family life. Nevertheless, they did want to share the responsibilities at home and saw their home as a mutual project in some ways. Wyatt offers this description: *“Yes, we try to help each other out. However, as a matter of fact, she pulls a bigger load than me. Anything else would be a lie. I’m so darned blind … if there’s a coffee cup on a workbench, I do not think about it, I just walk past it. While she sees everything … But then I tried. I have to lighten the burden some way, otherwise, I will not have a wife.”* Hence, this feeling of guilt also appears to be linked to the division of labor and the sacrifices needed to make family life work. However, no matter how hard they try, they do not feel they can compensate in their private life roles. This feeling was intensified, especially if their life partner had a daytime job while they were home “resting” because they had a day off in the middle of the week. Yet often their life partner would still not be satisfied with their efforts if they tried to contribute to the household chores. As Leo puts it: *‟…mostly try to sort of compensate for both my personal shortcomings and then the shortcoming of me working three shifts and maybe being away a bit, so that my wife does not have to do as much. And that makes me really go beyond myself to try to be there for my wife and my family and I do not know, it probably does not matter what I do, it does not really work.”* Female police officers also expressed guilt, but it was more directed at them not spending enough time with their children than not “helping out” enough at home.

Another aspect relating to support was that many male police officers described that having a life partner who was understanding toward their professional role was important if their life partner did not work in the police force themselves. Those that had life partners who were also police officers were relieved that their life partner could relate. This was an aspect only mentioned by the male police officers. As Leo puts it: *‟ …one reason why my relationship has lasted with my wife is probably because she is a police officer herself so she has some kind of knowledge or some understanding otherwise I would have probably been divorced by now I think (laughs)….”* The feeling of having an understanding life partner towards their professional role, seems to be important, at least for the male police officers. Furthermore, both men and women mentioned how talking to each other about everything, from minor details to major issues, even if you do not always agree on everything was important. The police officers emphasized that it had not always been easy and that they had also attempted to seek family counseling to improve communication in their partnership. Hence, many mentioned communication and support in various forms as important for taking on the challenges that exist when building strong life partner relationships, especially during difficult times, such as when struggling within parental roles or experiencing life crises. Another aspect was the importance of having someone they could talk to about their professional role if they needed to. Thus, obtaining social and emotional support was sometimes sought from family members. This however included a schism between roles because they were unable to fully describe what they have been through at work during the day, either due to the confidentiality of the job or because they did not want to horrify or appall their life partner. This meant that they did not always get what they needed when searching for emotional support from their life partner. Nevertheless, they were still clear about the fact that if it became too much to handle for their life partner, they should show respect and seek support from their workplace. Ethan describes this as *“… I just need to be able to tell someone … and it becomes natural that you want to talk about it at home. But … then I do not because she kind of does not want to know everything … she likes to listen … but she does not want to hear about horrible things … she cannot handle that well.”* Hence, even though they are clear on how they should handle this conflict between their professional role and private life roles, they are still left with emotional strain.

## 5. Discussion

Overall, the role balance of the uniformed police officers in this study is a multifaceted issue that encompasses finding harmony between professional and private life roles, managing priorities while working shifts, as well as trying to build strong relationships through emotional and social support. It sheds light on the complexities that police officers face struggling to maintain a balanced and fulfilling life both on and off duty. The police officers describe their everyday life and the challenges they experience in both their professional and private life roles. Police-specific challenges include both enrichments and conflicts when having a family while working as a uniformed police officer. As an example, one conflict is their hypervigilance when in private life roles, while an enrichment is being able to assess risks to know when there is actual danger or not. Another more general issue identified in the study was gender-patterned role conflicts. Both the female and male police officers in the study felt that the women in their respective relationships were pulling the heavier load regarding household and children. In addition, preference was given to the men’s professions in the relationships, which seemed to cause guilt. Furthermore, resulting in a role conflict based on emotional strain for both men and women in the study.

Our study revealed that police officers feel conflicted about their roles and struggle to balance their professional and private life commitments. Thus, maintaining relationships when shifts clash, overtime is a reality, and there is no social network to fall back on were contentious issues that our study identified. The answers to this problem should not only come from the police officers themselves but also from their employers and local governments, for example, by offering night care for children. This is especially important if the police officers are single parents. Our findings concur with another Swedish study on single working mothers who work shifts. They have been found to have frequent difficulties matching preschool opening hours with their employment schedules (Roman, 2017). Our study found that the police officers also needed a family-friendly workplace. Supportive workplace examples included flexible solutions regarding shift work. However, it was not possible for everybody. Consequently, the findings indicate that to be able to work as a uniformed police officer, a supportive social network is needed. This is in line with another Swedish study, where Alsarve (2017) found access to social support as a single working parent especially crucial (Alsarve, 2017). Hence, despite all the legislations and family policies, promoting gender equality in workplaces in Sweden, not all aspects seem to be implemented at different police departments in Sweden, as seen in our study. Although our sample is small, we can still find gender inequalities, where, for example, childcare for shift workers is regarded as insufficient. Hence, the findings highlight the stress that arises between roles because of time demands, emotional strain, or behavioral requirements for key roles in life, according to Evans et al. (2014).

The consequences of role balance are also important when raising awareness of uniformed police officers’ work-life balance, as professional roles tend spilling over into private life domains. In our study, there were differences in how role conflict was perceived based on gender. Thus, if Police authorities want to keep both male and female police officers in uniforms, providing for instance a family-friendly work environment, with flexible schedules for parents is important. Furthermore, it would be important to investigate the different types of role conflict strain experienced by male and female police officers. Moreover, to determine whether work-life conflict is experienced similarly or differently between genders, more research should be done using our findings. For example, a previous study on work–family conflict has revealed that while both men and women may experience the same amount of work–family conflict, there is also a gender suppression effect indicating that women work fewer hours or leave their jobs, to adjust for their work–family conflict, while men generally will not. This results in the same levels of work–family conflict for both genders (Young et al., 2023). Grönlund and Öun (2018) also found that women make choices to keep work–family conflict at a bearable level while at work, often avoiding family-unfriendly work conditions. The study also controlled for female-coded and male-coded professions, such as police officers (Grönlund and Öun, 2018). Hence, more research is needed also within the domain of work–family conflict of Swedish police officers, especially focusing on gendered patterns within the police force, and how it affects police officers’ private life domains.

According to Rawski and Workman-Stark (2018), there are four elements of a masculinity contest culture—showing no vulnerability, being strong and showing stamina, putting work first, and having the desire to hurt others to thrive in one’s own right—and these have also been studied in relation to police organizations in other countries (Rawski and Workman-Stark, 2018). Thus, working in a male-dominated organization may also be one of the reasons why the police officers preferably searched for emotional support from their life partners. This should however be studied further. But a Norwegian study on help-seeking behavior within the police service showed that less than 10% of police officers experiencing depression and suicidal ideation had contacted a psychologist or psychiatrist. Instead, they sought help from physiotherapists or chiropractors (Berg et al., 2006). Although, we were unable to find evidence to support all of the four elements of masculinity contest culture according to Rawski and Workman-Stark (2018), we also found another aspect “putting work first”. Hence, it is reasonable to assume that this phenomenon also exists among Swedish police officers, despite Sweden’s efforts to advance gender equality and its ranking as one of the most gender-equal nations in the European Union (Gender Equality Index 2020 Report, 2020).

The male police officers in the study also expressed being stressed that they were not doing enough to participate in parenting and domestic work. This is in line with Caroly (2011) who regards the division of labor within a male environment to be marked by workload regulations based on sexual stereotypes, whereas in female environments, like nursing, collective regulations allow schedule adjustments to meet domestic needs (Caroly, 2011). Consequently, despite sunshine examples found in our study, within a hierarchical and inflexible structure, adjustments to schedules are difficult to achieve. This implies a social injustice in a male-dominated context, which contributes to maintaining a gendered division of labor, also visible in our study. These gender-related factors may also be amplified for women in male-dominated professions, particularly if their life partner is also a police officer. Regardless, our results need further investigation on a larger scale. According to Guppy et al. (2019), gender behavior in households is also changing towards a more gender-friendly dynamic. This is in part due to changes in men’s behavior, and involvement in parenting (Guppy et al., 2019). Thus, this could be another reason for the male police officers feeling guilty for not sharing the household workload enough, and that their careers are prioritized before their life partners. It could also be the reason for the female police officers’ feel of discontent. Hence, guilt is a way of addressing gender issues in life-partner relationships, and the attempt to shoulder an equal share of the load. However, the gender-related issues found in this study should be studied on a larger scale since they could be related to health issues. It is especially important, since according to Harrysson and Elwér (2017) our society’s gender system affects our health negatively, and previous studies show that females have been found to experience more sick leave than men (Harrysson and Elwér, 2017). According to Grönlund and Öun (2020), having a parent-friendly schedule and being able to share the workload at home is important, it would also alleviate some of the issues raised in our study regarding emotional stress of the police officers. Particularly how sharing of childcare would reduce women’s stress. In our study we discovered a tendency for women to experience a double-work burden with both paid and unpaid work, which the male police officers are also aware of in their respective relationships and try to mitigate. For example, the female police officers were concerned with not having enough time with their children. The male police officers instead indicated that they worried about financial issues and their life partners carrying a heavier household burden than them. According to Duxbury and Higgins (2012) life partners of police officers tend to spend more time on domestic care than they do (Duxbury and Higgins, 2012). Moreover, studies on role balance in heterosexual relationships support our findings of gender differences in the data (Marks et al., 2001). It is also in line with Connell (2021) suggesting that there is a gendered division between paid and unpaid work within the gender system in our society, indicating unpaid work at home being more a female responsibility, whereas paid work is more of a male concern (Connell, 2021). However, as a result, this experience left the male police officers with feelings of guilt towards their life partners because of not spending enough time on household chores. The female police officers were not content either due to spending too much time on household chores, instead of with their children.

The issue of role balance as a uniformed police officer also comes to a head with some of the police-specific aspects. One role-conflicting example is their hypervigilance, which comes with their professional role. This hypervigilance seems to cause a behavioral strain for the police officers, indicating a between-role conflict, where spare-time activities in private life is avoided. Conflicts between work and family life have according to Nilsen et al. (2017) been found to be associated with later sickness absence, indicating the need to mitigate the risks of conflicts between professional and private life roles (Nilsen et al., 2017). The finding in this study is also in line with previous research, where police officers have been found to worry about their family’s safety, for example checking for bombs under cars (Sundqvist et al., 2021). Worrying about the well-being of their families while also juggling more common life demands, may place additional emotional strain on the police officers, which requires further research beyond this study. However, being concerned with risks could also cause role enrichment among the police officers. Thus, the knowledge of risk assessment was passed over from their professional role to their private life roles resulting in emotional relief instead of stress. Another police-specific aspect causing both conflict and enrichment between roles is when the police officers due to their professional role are left with an emotional strain due to job tasks. By searching for emotional support in their private life this strain can however at times be eased. Also, it may be temporarily lifted by having children, since at home, everything focuses on them. Another example of both role conflict and enrichment is how shift work may cause conflicts between roles, but it can also ease role strain. For example, the police officers felt that shift work allowed them to have more time with their families. According to Evans et al. (2014), when skills, resources, and energy are shared between various roles through enrichment, positive experiences in one role may help the person avoid negative experiences in another (Evans et al., 2014).

## 6. Limitations

According to Elo et al. (2014), the trustworthiness of a qualitative content analysis should be regarded from collecting data to finalizing the study in writing. For this study, dependability issues need to be raised regarding sampling strategies and might be one of the study’s important shortcomings since it largely includes Caucasian heterosexual couples, possibly limiting the variety of perspectives. Although the data over time might be stable, the other aspect of dependability, namely stability under different conditions, might be impossible to prove. For the same reasons, the transferability of results might also be an issue. Thus, studying the challenges in the intersection of police officers’ professional and private life roles should be conducted on a larger scale to include more diversity in the future.

The intention was to utilize semi-structured interviews when gathering data for this study. The choice was made to concentrate on the participants’ narratives as data collection went along, with a final check to make sure all subjects had been covered. While changing methods might seem like a limitation, it should, according to the credibility of qualitative content analysis, instead be seen as a strength since it also reflects self-awareness of the researchers. Transcripts were also read several times to obtain a sense of the whole, as well as keeping notes of emotional expressions in the transcripts to ensure the transmission of intrinsic meaning and strengthen trustworthiness.

To increase credibility, the findings were presented and discussed with other researchers, and feedback was solicited from those not involved in the study. To increase the conformability of the data, the entire author group reviewed the final analysis and checked that quotations within a theme were consistent with the theme itself. Rich descriptions of findings, illustrative quotes, as well as quotes from all participants, were used to demonstrate the grounding of the data. Defining the study context, participants, and settings as clearly as possible should also strengthen the issue of transferability. Also, to gain a researcher’s perspective on the data and strengthen methodological integrity, as well as the credibility and dependability of the results, each author contributed their expertise, linking existing literature to the themes and expanding on similarities and differences identified in the data. The authors all have different expertise, thus, throughout the entire process, different competencies and perspectives were brought to the analysis.

## 7. Conclusion and practical implications

This study sheds light on how police officers balance their professional roles with personal responsibilities as life partners and parents. The study advances our knowledge of the extra hardships that police officers experience because of their job duties and how this impacts their personal lives. For instance, feeling torn between roles, experiencing the overshadowing effects of their demanding profession, being constantly alert and vigilant, and dealing with feelings of guilt. However, the study also highlights that the police officer role can positively impact their private life roles in various ways, such as how being a police officer can bring enrichment and fulfillment to their personal lives. The results from this study may also apply to other male-coded contexts, such as firefighters, or military personnel, where the consequences of work are also a challenge to combine with family life. Furthermore, the study touches upon other wider-ranging challenges like working shifts, which pose a crucial question about how difficulties, particularly those arising from gender inequality, can be mitigated within police forces. These findings have practical implications for police officers, who must be able to successfully balance their personal and professional lives while taking care of a family. For instance, encouraging a gender-inclusive workplace would be crucial, where both male and female police officers have the option of continuing to work as uniformed police officers even after starting a family or becoming single parents. As well as to do so in good health. Hence, the findings may also be helpful in clinical practice when working with police officers’ health or the well-being of police officers’ families.

## Data availability statement

The datasets presented in this article are not readily available because of the sensitive nature of the data, which needs special ethical considerations. Thus, the data cannot be shared with a third party. Requests to access the datasets should be directed to elin.granholm@umu.se.

## Ethics statement

The studies involving human participants were reviewed and approved by Swedish Ethical Review Authority. The patients/participants provided their written informed consent to participate in this study.

## Author contributions

EGV and UN planned the study. EGV conducted and analyzed the interviews, while UN provided feedback on the themes and data at several points in time. KG and MG also commented on the analysis as the process went by EGV, UN, MG, and KG provided feedback regarding the naming of the themes. EGV constructed the first draft of the manuscript, while UN, MG, and KG provided feedback on different parts of the manuscript. All authors contributed to the article and approved the submitted version.

## Funding

This study is part of a Ph.D. research project funded by the Department of Community Medicine and Rehabilitation, Umeå University, and the Kempe Foundation.

## Conflict of interest

The authors declare that the research was conducted in the absence of any commercial or financial relationships that could be construed as a potential conflict of interest

## Publisher’s note

All claims expressed in this article are solely those of the authors and do not necessarily represent those of their affiliated organizations, or those of the publisher, the editors and the reviewers. Any product that may be evaluated in this article, or claim that may be made by its manufacturer, is not guaranteed or endorsed by the publisher.
